# Effect of COL4A1 Expression on the Survival of Neoadjuvant Chemotherapy Breast Cancer Patients

**DOI:** 10.1155/2020/5209695

**Published:** 2020-05-14

**Authors:** Shin-Mae Wang, Po-Ming Chen, Yu-Wen Sung, Wei-Chieh Huang, Hung-Sen Huang, Pei-Yi Chu

**Affiliations:** ^1^Department of General Surgery, Show Chwan Memorial Hospital, Changhua 500, Taiwan; ^2^Research Assistant Center, Show Chwan Memorial Hospital, Changhua 500, Taiwan; ^3^Chinese Medical Research Center, China Medical University, Taichung 404, Taiwan; ^4^Department of Obstetrics and Gynecologics, China Medical University Hospital, Taichung City, Taiwan; ^5^School of Medicine, College of Medicine, Fu Jen Catholic University, New Taipei 242, Taiwan; ^6^Department of Pathology, Show Chwan Memorial Hospital, Changhua 500, Taiwan; ^7^Department of Health Food, Chung Chou University of Science and Technology, Changhua 510, Taiwan

## Abstract

Optimal therapy for each patient depends on their subtype, anatomic cancer stage, gene status, and preferences. Neoadjuvant chemotherapy-treated tumors have shown attenuated tumor growth, but the therapy cannot completely reduce tumor cell dissemination to blood stream and distant metastasis. Though it has been indicated that the protein of the collagen type IV alpha 1 (COL4A1) gene is induced by p53 to inhibit angiogenesis and tumorigenic activity in cancer cells, its prognostic significance in breast cancer (BC) patients has not yet been fully elucidated. We analysed 206 BC and fresh paired-match adjacent normal breast tissue from tissue microarrays (TMAs) and COL4A1-stained TMAs using immunohistochemistry. These were used to evaluate COL4A1 expression in BC and to analyse the relationship between this expression and clinicopathological factors and prognosis. The expression of the COL4A1 protein was significantly higher in normal adjacent tissue than in the tumor tissues of BC (*P* < 0.0001). The low COL4A1 expression of the BC patients had decreased metastasis incidence ratio than those exhibiting high COL4A1 expression (*P*=0.034). Low COL4A1 expression in the tumor cells of BC patients was found to significantly reduce the overall survival (OS) and relapse-free survival (RFS) rates of neoadjuvant chemotherapy patients (*P*=0.047 and *P*=0.025, respectively). We also validated the results to ensure their consistency with a web server program for survival analysis from the Cancer Genome Atlas (TCGA) database (*P*=0.057). Additionally, COL4A1 expression was positively correlated with p53 expression (*P*=0.00076). Thus, we present clinical evidence that COL4A1 expression can be used as a biomarker of better prognosis of BC patients receiving neoadjuvant chemotherapy.

## 1. Introduction

Breast cancer (BC) is the most frequently diagnosed cancer and the second leading cause of cancer-related mortality after lung cancer among women worldwide [1]. Breast cancer in Asian populations is characterized by an early-onset and relatively younger median age upon diagnosis than in Western populations [2]. For invasive breast cancer, similar longitudinal age-specific incidence probabilities along with converging incidence rate ratios (IRRs) reveal that the age effects are more similar among Asian and Western populations [3]. Human mammary glands have a highly branched network of epithelial tubes, embedded within the breast which undergoes changes in size, shape, and function to puberty, pregnancy, and lactation, in response to steroid hormone and growth factor receptor signalling. However, the aberrant signalling pathways by which they contribute to breast carcinogenesis and breast cancer type can be classified into various subtypes based on the expression of the estrogen receptor (ER), progesterone receptor (PR), and human epidermal growth factor 2 (HER2) patterns, which implied significant overall and disease-free survival advantages [4–6]. TP53 (p53) is the most frequently mutated gene associated with greater disease aggression and worse overall survival [7]. Although mutated in 30–35% of all cases, p53 is mutated in approximately 80% of triple-negative (TN) tumors (i.e., tumors negative for ER, PR, and HER2) [8]. So far, mutant p53 cannot be recommended as a prognostic or therapy predictive biomarker in BC, and some studies had until recently investigated it as a potential target for BC treatment [7, 8]. The genetic and epigenetic changed in p53 have been identified in regulators of p53 activity as well as in some downstream transcriptional targets of p53 in BC that express wild-type p53 [7, 8].

p53 is not only a well-known activator of apoptosis or cell cycle arrest in response to cellular stress or DNA damage stemming from protection of genomic stability, it also enhances antiangiogenic effects, monitors tumor inflammation and immune response, and inhibits metastases [9, 10]. COL4A1 is a major antiangiogenic gene induced by p53 in human adenocarcinoma cells, and p53 directly activates the transcription of the COL4A1 gene by binding to an enhancer region 26 kbp downstream of its 3′ end [11]. COL4A1, the collagen IV molecule, is 400 nm long and involved in cell interactions with cells, possessing two specific recognition sites for the integrins alpha 1 beta 1 and alpha 2 beta 1 [12].

We analysed 206 surgical specimens from patients with breast cancer and adjacent normal tissue using IHC staining with a specific antibody against COL4A1 and evaluated the correlation between the clinical outcomes and the IHC scores of COL4A1. We further investigated the correlation between COL4A1 expression and long-term OS and RFS in patients with breast cancer via Kaplan–Meier analysis.

## 2. Materials and Methods

### 2.1. Patients

Contralateral primary breast tumor and adjacent normal breast tissues were acquired from 206 BC patients receiving surgical resection at Changhua Show Chwan Memorial Hospital from March 2011 to January 2017. Computed tomography (CT) was used for staging in the breast cancer patients prior to surgery. The diagnosis parameters and clinical outcomes were recruited until the patient's death, censorship, or loss to follow-up. For each patient, representative tissue cores of the BC tumor section as well as the adjacent normal section were carefully collected and made into tissue microarray. The age of all patients was between 30 and 95 years (mean ± SD 54.36 ± 11.62). Clinical parameters and overall survival data were collected from a cancer registry system at the Changhua Show Chwan Memorial Hospital. Survival time was defined to be the period of time from the date of primary surgery to the date of death. The median overall survival of all breast cancer patients was 1485 days. During this survey, 25 patients died and 46 relapsed. On the basis of the follow-up data, 42 patients exhibited tumor metastasis, with the metastasis sites, including bone, lung, liver, chest wall, breast, and lymph node. There were 42 metastasis cases: three in the lymph node, 19 in the breast, four in the lung, one in the pleura, one in the liver, seven in the bone, one in the chest wall, one in the chest skin, one in the breast and lung, one in the liver and bone, one in the breast and liver, one in multiple organs, and one in the bone and abdomen. This project was approved by the Ethics Committee of the Institutional Review Board of Show Chwan Memorial Hospital (IRB no. 1060407).

### 2.2. Immunohistochemistry and Scoring

Immunohistochemistry (IHC) staining was used to evaluate COL4A1 protein expression. The COL4A1 antibody (NB120-6586) was purchased from Novus Biologicals (Novus Biologicals, Littleton, CO, USA). Please also have a look at https://www.novusbio.com/products/collagen-iv-alpha-1-antibody_nb120-6586#PublicationSection. The COL4A1 antibody (NB120-6586) has been tested in human, mouse, rat, and bovine and applied to immunocytochemistry (ICC), immunofluorescence (IF), immunohistochemistry (IHC-frozen sections and paraffin-embedded sections), enzyme-linked immunosorbent assay (ELISA), and western blot (WB). Previously described IHC evaluation and protocol were used to obtain score [13]. The mean signal scores were evaluated independently by the two pathologists who were blinded when assessing the samples. Immunostaining scores were defined as the cell staining intensity (0 = nil; 1 = weak; 2 = moderate; and 3 = strong) multiplied by the percentage of labeled cells (0% to 100%), leading to scores from 0 to 300. The mean of score of signals was evaluated independently by the two pathologists. The median IHC staining score (75) was used as the cutoff point for the dichotomization of COL4A1. A score greater than 75 was defined as “high” immunostaining, whereas a score of less than or equal to 75 was defined as “low.”

### 2.3. Statistical Analysis

The association between COL4A1 expression and the clinical and pathological parameters was calculated using chi-square and paired-sample *t*-tests, and survival curves were plotted using the Kaplan–Meier method and compared using the log-rank test. Cox's proportional hazard regression model was used to analyse the association between the variables and survival data. *P* < 0.05 was considered to indicate a statistically significant difference. SPSS 18.0 (SPSS, Inc., Chicago, IL, USA) was used for all statistical analyses.

### 2.4. Web Server Survival Analysis

The survival analysis of COL4A1 expression of in this study was performed using the web server for the Kaplan–Meier plots from the Cancer Genome Atlas (TCGA) datasets by autoselecting the best cutoff values between the lower and upper quartiles into high and low expression groups which are computed in all stages, gender, race, and mutation burden.

Please have a look at https://kmplot.com/analysis/index.php?p=service&cancer=breast#. The gene chip data sources for the databases include GEO, EGA, and TCGA. The primary purpose of the tool is the meta-analysis-based discovery and validation of the survival biomarkers. All cutoff values between the lower and upper quartiles, as well as the best performing threshold, were used as a cutoff.

## 3. Results

### 3.1. COL4A1 Expression Is Significantly Higher in Normal Tissues

**Figure 1:**
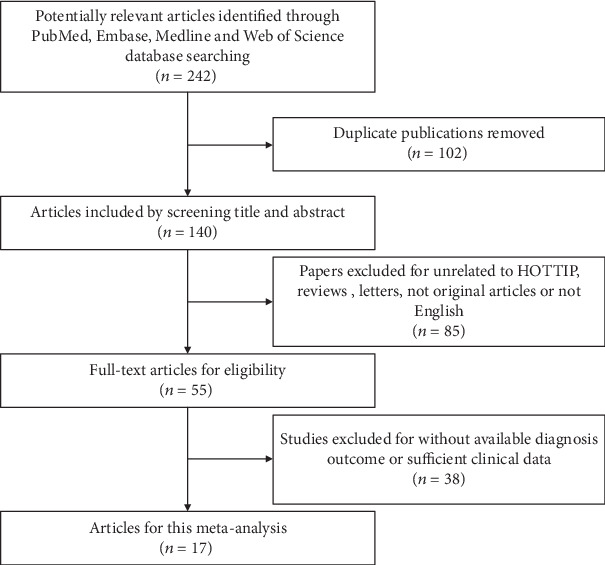
COL4A1 expression in adjacent normal and tumor breast tissue of BC patients. (a) Representative high COL4A1 immunostaining results in adjacent normal breast tissue. (b) Representative high COL4A1 immunostaining results in breast cancer tissue. (c) *t*-test for COL4A1 levels was compared in tumor and pair matched nontumor breast tissues of BC patients.

We enrolled 206 BC patients to estimate COL4A1 protein detected using immunohistochemistry in 206 paired tumor and adjacent normal breast tissue. Representative results are shown in Figures 1(a) and 1(b), and COL4A1 expression was observed in the cytoplasm of the tumor and paired adjacent normal tissues. COL4A1 was expressed at higher levels in the breast cancer tissues compared to the 206 pairs of adjacent normal tissue (*P* < 0.0001, Figure 1(c)). Of the 206 pairs, the expression level of COL4A1 in the tumor tissue was higher than in the normal tissue from the 161 pairs (161/206, 78%).

### 3.2. COL4A1 Expression Is Positively Correlated with Tumor Metastasis

As shown in Table 1, low COL4A1 expression in tumors was significantly associated with tumor metastasis (*P*=0.034), but no significant association was found in those over aged 65 (*P*=0.421), with late-stage tumors (*P*=0.058), with positive ER expression (*P*=0.092), with positive PR expression (*P*=0.257), positive HER2 expression (*P*=0.647), or who received neoadjuvant chemotherapy (*P*=0.742).

### 3.3. Age, Stage, ER, PR, Metastasis, and Neoadjuvant Chemotherapy Characteristics Are Correlated with Overall Survival (OS) in BC Patients

**Figure 2:**
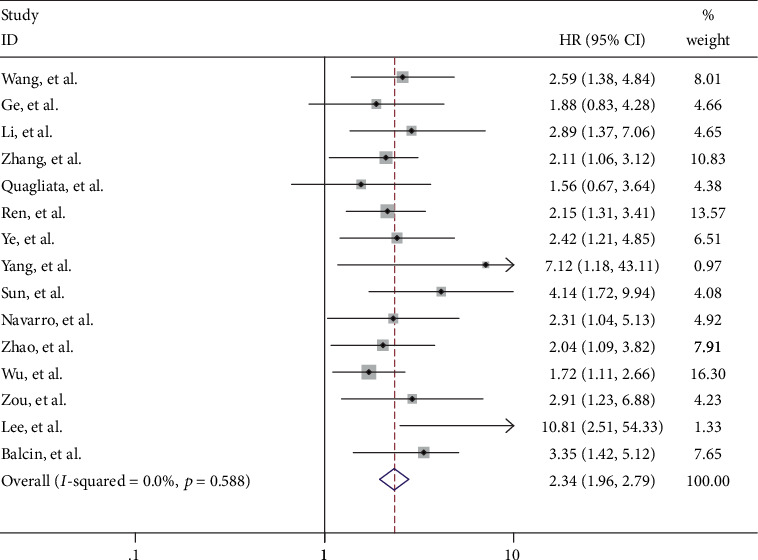
Kaplan–Meier analysis of different characteristics for patients. (a) Overall survival estimates for age. (b) Overall survival estimates for tumor stage. (c) Overall survival estimates for ER. (d) Overall survival estimates for PR. (e) Overall survival estimates for HER2. (f) Overall survival estimates for tumor metastasis. (g) Overall survival estimates for neoadjuvant chemotherapy. (h) Overall survival estimates for COL4A1 expression.

Kaplan–Meier survival curves further showed that late-stage tumors (*P* < 0.001), ER-positive tumors (*P*=0.0005), and PR-positive tumors (*P*=0.012) were associated with poor survival probability (Figure 2). Furthermore, patients aged 65 and above (*P* < 0.001), patients with metastasis (*P* < 0.0001), and patients who received neoadjuvant chemotherapy (*P* < 0.0001) exhibited poor survival probability (Figure 2).

### 3.4. Age, Stage, Metastasis, and Neoadjuvant Chemotherapy Characteristics as Independent Prognosis Factors in BC

**Figure 3:**
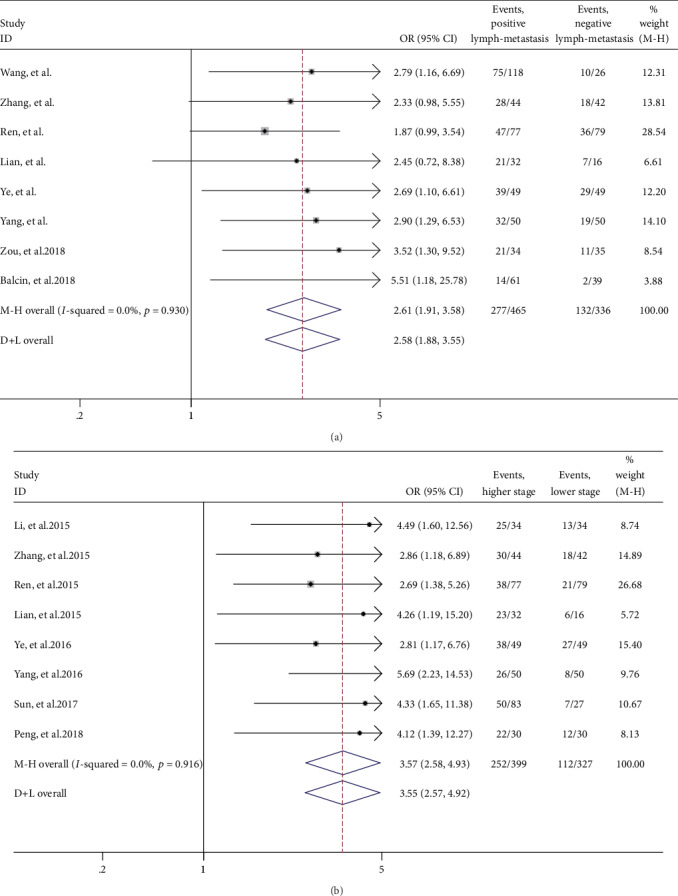
Cox regression analysis for the influence of parameters and COL4A1 on overall survival in all BC patients. Statistical tests were two sided. HR = hazard ratio and CI = confidence interval.

We further examined whether the clinical parameters could be the independent prognosis factors in BC patients. We performed Cox's regression analysis with these factors in order to estimate the independent effect of the OS of BC. In the multiple univariate analysis, age, stage, metastasis, and neoadjuvant chemotherapy status were predictive of poor overall survival (*P* < 0.001, 0.001, 0.001, and 0.002, respectively; Figure 3).

### 3.5. COL4A1 Expression as a Better Prognosis for Overall Survival (OS) and Relapse-Free Survival (RFS) in BC Patients Who Received Neoadjuvant Chemotherapy

**Figure 4:**
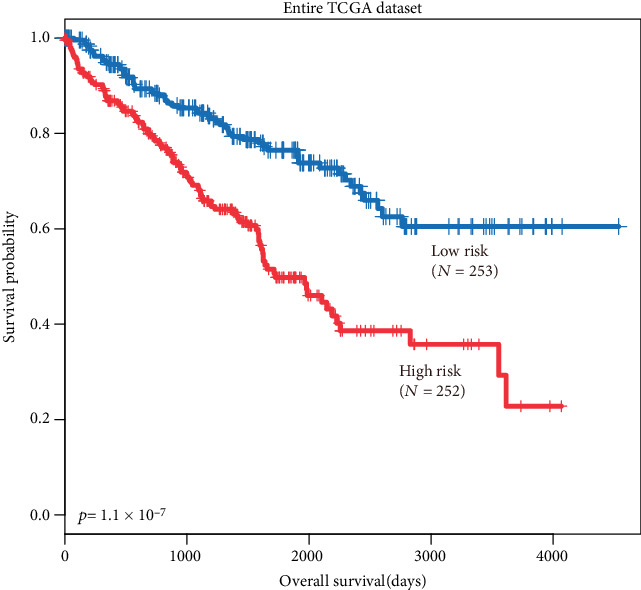
Kaplan–Meier analysis for the influence of COL4A1 expression, stage, and age on overall survival (OS) and relapse-free survival (RFS) in BC patients with neoadjuvant chemotherapy. (a) Overall survival estimates for COL4A1 expression. (b) Overall survival estimates for stage. (c) Overall survival estimates for age. (d) Relapse-free survival estimates for COL4A1 expression. (e) Relapse-free survival estimates for stage. (f) Relapse-free survival estimates for age.

In BC patients who received neoadjuvant chemotherapy but not without received neoadjuvant chemotherapy, we found that those exhibiting a high expression of COL4A1 had longer OS and RFS periods than those exhibiting a low expression as determined via Kaplan–Meier analysis (*P*=0.047 and *P*=0.026, Figures 4(a) and 4(b)). As shown in Table 2, the low expression of COL4A1 was positively associated with the late-stage cancer (III and IV) (*P*=0.046), but there was no association between COL4A1 expression and age (*P*=0.688). 42 patients with late-stage tumor had a lower overall survival and relapse-free survival rate compared with early-stage cancer (*P* < 0.0001, Figure 4). The older patients (≧65 years old) had a lower overall survival rate than the younger patients (<65 y/o) (*P* = 0.026, Figure 4), but there was no significant in the relapse-free survival rate.

### 3.6. COL4A1 mRNA Expression Is Positively Associated with p53 Expression and Identification of COL4A1 mRNA Expression in terms of Survival Rates of BC Patients Who Received Neoadjuvant Chemotherapy in the Web Server

**Figure 5:**
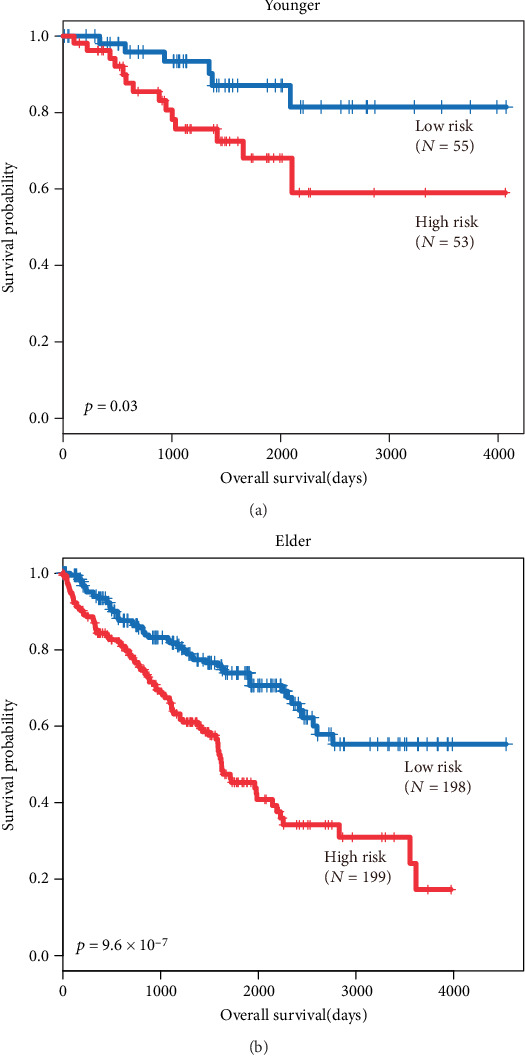
The web server for the COL4A1 expression analysis. (a) Pearson's correlation for p53 and COL4A1 expression. (b) Kaplan–Meier analysis of the influence of and COL4A1 expression on overall survival (OS) in BC patients with neoadjuvant chemotherapy.

In terms of correlation, COL4A1 mRNA expression was positively associated with p53 mRNA expression (*P*=0.00076, Figure 5(a)) as assessed via a web server program, an enhanced web server for large-scale expression profiling and interactive analysis (http://gepia2.cancer-pku.cn/#correlation). The breast cancer mRNA database was searched to analyse the expression of COL4A1 mRNA in BC patients who received neoadjuvant chemotherapy and the effects on their overall survival. We found that low COL4A1 expression was associated with a poor prognosis for overall survival (OS) for breast cancer patients who received neoadjuvant chemotherapy (*P*=0.057, Figure 5(b)) (https://kmplot.com/analysis/index.php?p=service&cancer=breast).

## 4. Discussion

Neoadjuvant chemotherapy is a standard of strategy that is widely used for locally advanced and early breast cancer patients [14]. The varying roles of the different regimens used as neoadjuvant chemotherapy need to be investigated, as it is currently unclear which treatment regimen suits best [14]. Prior to the surgical removal of the tumors, patients receive neoadjuvant chemotherapy and can downstage tumors allowing breast-conserving surgery, rather than mastectomy and setting offers a valuable opportunity to monitor individual tumor response [15]. Prior to the surgical removal of the tumors, patients receive neoadjuvant chemotherapy, can downstage tumors allowing breast-conserving surgery, rather than mastectomy and setting which offers a valuable opportunity to monitor individual tumor response [15]. In 42 patients who received neoadjuvant chemotherapy, the expression level of COL4A1 in the tumor, as assessed by immunohistochemistry, was not associated with age (Table 2), but was highly positively correlated with stage status (*P*=0.046). Furthermore, the failure of neoadjuvant chemotherapy will make it more hospitable to resisted cancer cells upon their arrival at the distant sites due to change in the nontumor tissue microenvironment [16, 17].

The clinical characteristics showed that the positive expression of ER and PR tumors was associated with longer OS in BC patients (Figures 2(c) and 2(d)), but there was no significant correlation on OS by the Cox regression model (Figure 3). Indeed, triple-negative breast cancers (TNBCs), which account for approximately 15% of all breast cancer patients, are negative for ER, PR, and HER2 and exhibit poor prognosis and limited treatment options relative to other breast cancer subtypes [18]. The BC patients who received neoadjuvant chemotherapy have poor prognosis by Kaplan–Meier analysis and Cox regression that was because these patients had higher frequency of the late tumor stage (17/42, 41%) (Figures 2(g) and 3; Table 2).

In this cohort, metastasis sites including bone, lung, liver, chest wall, breasts, and lymph nodes that shorten the survival rate of BC patients were correlated with low COL4A1 protein expression (Table 1). Tumor metastasis correlated with worse prognosis, and COL4A1 expression in BC patients who received neoadjuvant chemotherapy could predict favourable OS and RFS as assessed via the Kaplan–Meier method (Figures 4(a) and 4(b)). COL4A1 downregulation in infertile human endometrium reduces endometrial epithelial cell adhesive capacity [18], which implied COL4A1 expression can inhibit primary tumor segregation and result in metastasis.

Several types of collagen have been identified to process antiangiogenic domains that can be released by proteolysis of the basement membrane (BM), a specialized form of the extracellular matrix [19, 20], and COL4A1 is a major transcriptional target of p53 [11]. We found COL4A1 expression contributed to the better prognosis in BC patients who received neoadjuvant chemotherapy.

The p53 protein rapidly accumulates in cells in response to chemotherapy, which is important for tumor suppression by p53, and implicit in the p53 induction-apoptosis pathway inhibiting tumor cells, especially in T-cell lymphomas, intestinal adenomas, and mammary tumors [20–24]. However, p53-induced COL4A1 function is still unknown in BC patients who received neoadjuvant chemotherapy. We further calculated the correlation between p53 and COL4A1 by GEPIA2 (*P*=0.00076, *R* = 0.1, Figure 5(a)) (http://gepia2.cancer-pku.cn/#correlation) and investigated the COL4A1 prognostic impact on BC patients who received neoadjuvant chemotherapy (*P*=0.057, Figure 5(b)) [25].

## 5. Conclusion

In conclusion, we proposed for the first time that COL4A1 could act as a prognostic marker of survival for BC patients who underwent neoadjuvant chemotherapy. Despite the modest sample size of the analysed BC samples, this study successfully provided the prognosis marker and therapeutic targets for BC patients who received neoadjuvant chemotherapy. Our results are helpful in the evaluation of BC patients who received neoadjuvant chemotherapy before the surgical removal of a tumor.

## Figures and Tables

**Table 1:** Relationship of clinical parameters with COL4A1 expression in 206 breast cancer.

Characteristics	No.	COL4A1 expression		*P* value
		Low (%) *N* = 88	High (%) *N* = 116	
*Age*				
<65	173	76 (44)	97 (56)	0.421
≥65	33	12 (36)	21 (64)	
*Stage*				
I, II	171	68 (40)	103 (60)	0.058
III, IV	35	20 (57)	15 (43)	
*ER*				
Negative	57	19 (33)	38 (67)	0.092
Positive	149	69 (46)	80 (54)	
*PR*				
Negative	77	29 (38)	48 (62)	0.257
Positive	129	59 (46)	70 (54)	
*HER2*				
Negative	33	13 (39)	20 (61)	0.647
Positive	173	75 (43)	98 (57)	
*Metastasis*				
No	164	64 (39)	100 (61)	0.034
Yes	42	24 (57)	18 (43)	
*Neoadjuvant chemotherapy*				
No	164	71 (43)	93 (57)	0.742
Yes	42	17 (40)	25 (60)	

**Table 2:** Relationship of age, stage, and COL4A1 expression in 42 breast cancer patients who received neoadjuvant chemotherapy.

Characteristics	No.	COL4A1 expression		*P* value
		Low (%) *N* = 17	High (%) *N* = 25	
*Age*				
<65	38	15 (39)	23 (61)	0.683
≥65	4	2 (50)	2 (50)	
*Stage*				
I, II	25	7 (28)	18 (72)	0.046
III, IV	17	10 (59)	7 (41)	

## Data Availability

The raw and derived data used to support the findings of this study are available from the corresponding author upon request.

## Abbreviations

COL4A1: Collagen type IV alpha 1
BC: Breast cancer
TMAs: Tissue microarrays
OS: Overall survival
RFS: Relapse-free survival
IRRs: Incidence rate ratios
ER: Estrogen receptor
PR: Progesterone receptor
HER2: Human epidermal growth factor 2
IHC: Immunohistochemistry.
